# Assessing health beliefs and patient delay among breast cancer patients in West Sumatra, Indonesia

**DOI:** 10.1016/j.mex.2026.104035

**Published:** 2026-07-07

**Authors:** Ricvan Dana Nindrea, Armaita, Ainil Mardiah, Long Chiau Ming, Ismeldi Syarief, Muhammad Syarif Khairullah Suryadharma Putra, Nadyne Falisha, Regita Ayu Cahyani, Tsania Faiza Mardhatillah, Novia Elsaputri

**Affiliations:** a Department of Medicine, Faculty of Medicine, Universitas Negeri Padang, Bukittinggi, Indonesia; b Department of Nursing, Faculty of Psychology and Health Sciences, Universitas Negeri Padang, Padang, Indonesia; c School of Pharmacy, Sunway University, Sunway City, Malaysia; d Datta Meghe College of Pharmacy, Datta Meghe Institute of Higher Education and Research (deemed to be University), Sawangi (M), Wardha, India; e Department of Surgical Oncology, Dr. Achmad Mochtar Hospital, Bukittinggi, Indonesia

**Keywords:** Health belief model, Patient delay, Breast cancer, Health-seeking behavior, Indonesia

## Abstract

Delays in receiving a breast cancer (BC) diagnosis and treatment continue to be a major public health concern, particularly in environments where financial status, cultural values, and religious beliefs have a significant impact on how individuals react to disease. However, there are still few established, culturally appropriate instruments for assessing Health Belief Model (HBM) constructs and comprehending how they contribute to patient delay. In this study, a protocol for evaluating HBM components and investigating their connection to patient delay among patients with BC in West Sumatra, Indonesia, is presented. A 31-item questionnaire encompassing six HBM domains: perceived risk, severity, benefits, barriers, self-efficacy, and cues to action was used to gather data from 250 BCE patients in this cross-sectional study. Exploratory factor analysis revealed five meaningful latent constructs, and one poorly performing item was removed to strengthen the instrument. Good internal consistency was shown by the final tool (Cronbach's α: 0.809–0.871). Associations between HBM components and patient delay were then examined through logistic regression using crude and adjusted models. The protocol proved practical and feasible in a community setting.

## Graphical abstract

**Figure:**
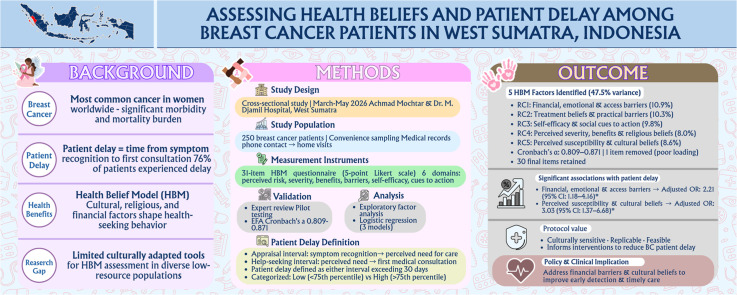

## Specifications table

**Table:** 

**Subject area**	Medicine and Dentistry
**More specific subject area**	Oncology; Cancer screening; Health behavior
**Name of your protocol**	A protocol for assessing health beliefs and patient delay among breast cancer patients in West Sumatra, Indonesia
**Experimental design**	A cross-sectional study conducted from March to May 2026 in West Sumatra, Indonesia. A total of 250 BCE patients were recruited using convenience sampling. Data were collected using a validated 31-item HBM questionnaire covering six domains: perceived risk, severity, benefits, barriers, self-efficacy, and cues to action, administered by trained interviewers following a standardized protocol. Exploratory factor analysis identified five latent constructs, and logistic regression was applied to examine associations between HBM components and patient delay.
**Trial registration**	Not applicable
**Ethics**	The Ethics Committee of Dr. M. Djamil Hospital in Padang, Indonesia, approved this study (No. DP.04.03/D.XVI.10.1/255/2026). All participants were properly informed about the study and gave signed consent to participate before any data were collected.
**Value of the Protocol**	• Brings together culturally sensitive HBM instruments with robust psychometric properties, making them suitable and meaningful for use among BC patients. • Sheds light on how particular health beliefs shape a patient's decision to seek timely BC diagnosis and treatment. • Generates practical, evidence-based insights that can guide the development of targeted interventions to help BC patients seek care without unnecessary delay.

## Background

Breast cancer remains the most commonly diagnosed cancer among women worldwide, accounting for a substantial proportion of cancer-related morbidity and mortality [1]. One of the most critical yet overlooked determinants of treatment outcomes is patient delay, defined as the time between when a person first notices their symptoms and when they finally seek medical attention [[2], [3], [4]]. When this gap is prolonged, patients are far more likely to be diagnosed at an advanced stage, leaving them with fewer treatment options and a significantly lower chance of survival. Despite growing awareness, patient delay remains a persistent and deeply rooted challenge, particularly in low- and middle-income countries where cultural values, religious beliefs, and financial hardships continue to shape how and when people choose to seek care [5,6].

The Health Belief Model (HBM) offers a well-established and widely recognized theoretical framework for understanding how people make decisions about their health [7]. Its core constructs, namely perceived susceptibility, severity, benefits, barriers, self-efficacy, and cues to action, have been extensively used to help explain why individuals delay seeking cancer screening and treatment [8,9]. However, the extent to which each of these components contributes to patient delay is far from uniform and tends to vary considerably depending on the cultural context. In communities where fatalistic outlooks, strong religious convictions, and financial difficulties are part of everyday life, these constructs can take on very different meanings and interact in ways that conventional, one-size-fits-all instruments are simply not equipped to capture [10,11].

Despite the clear importance of understanding these beliefs, the way HBM constructs are measured in BC patients remains frustratingly inconsistent across studies. Many existing studies continue to rely on instruments that were originally developed in high-income settings and have never been properly adapted to fit different cultural realities, raising serious questions about their validity and usefulness in diverse populations [12,13]. Furthermore, few studies have taken a comprehensive approach by examining multiple HBM components together in relation to patient delay, and attempts to use exploratory factor analysis to uncover culturally meaningful latent constructs remain remarkably rare [14,15].

There is therefore a pressing need for a systematic, culturally grounded protocol that can reliably assess HBM components and their relationship with patient delay. Such a protocol would generate robust and replicable evidence to help identify belief-related barriers that can realistically be changed, support the development of behavioral interventions that truly resonate with patients, and provide a solid foundation for health policies aimed at shortening the time between symptom recognition and treatment among BC patients.

This study differs from previous research by developing a culturally tailored and validated HBM instrument to assess breast cancer patient delay among women in West Sumatra, Indonesia. Rather than relying on tools from other contexts, this protocol captures local cultural, religious, fatalistic, financial, emotional, and access-related influences on care-seeking behavior. It also integrates exploratory factor analysis and logistic regression to identify relevant belief constructs and determine which factors are most strongly linked to delay. Thus, this study provides a context-specific, psychometrically sound, and reproducible approach to understanding and reducing breast cancer patient delay.

This study aims to develop and validate a protocol for assessing Health Belief Model constructs and patient delay in seeking BC diagnosis and treatment among breast cancer patients in West Sumatra, Indonesia.

## Description of protocol

This protocol was designed to assess health beliefs and patient delay among BC patients in clinical settings in West Sumatra, Indonesia.

### Study design and setting

This study employed a cross-sectional design and was carried out from March to May 2026 at Achmad Mochtar Hospital, Bukittinggi City, and Dr. M. Djamil Hospital, Padang City, West Sumatra, Indonesia. These hospitals were purposively selected as the study sites given their diverse patient population, encompassing individuals from a wide range of socioeconomic backgrounds and varying levels of access to healthcare services. This diversity not only enriched the quality of the data collected but also strengthened the generalizability and replicability of the protocol in other clinical settings facing similar challenges.

### Study population and sample size calculation

The study population comprised BC patients receiving care at Achmad Mochtar Hospital, Bukittinggi, and Dr. M. Djamil Hospital, Padang City, during the study period from March to May 2026. The sample size was determined using a standard formula for proportions, assuming a 95% confidence interval, a hypothesized prevalence of 20% (drawn from the estimated prevalence of patient delay reported in prior literature [16]), and a 5% margin of error, which yielded a minimum required sample of 250 patients. Convenience sampling was used, with eligible patients first identified through medical records at Achmad Mochtar Hospital and Dr. M. Djamil Hospital covering the same study period. Interviews were then arranged by reaching out to patients via telephone numbers available in their medical records. Where patients could not be contacted by phone, trained research assistants conducted home visits to ensure sufficient participation and reduce the likelihood of loss to follow-up. To be included in the study, patients were required to have a confirmed BC diagnosis, be aged 18 years or older, and be able to communicate verbally in Bahasa Indonesia. Patients were excluded if they had a history of other malignancies, were currently receiving psychiatric treatment, or were unable to provide informed consent owing to cognitive impairment.

### Measurement instruments

Patient delay was measured by examining two distinct time intervals. The first, known as the appraisal interval, captured the time that elapsed from when a patient first noticed her symptoms to when she felt the need to seek care. The second, the help-seeking interval, captured the time between that perceived need and her first actual consultation with a healthcare provider. Together, these two intervals made up what is referred to as the patient interval. A patient was considered to have experienced delay if either of these intervals exceeded 30 days [3,4,9,17].

HBM constructs were measured using a 31-item questionnaire organized around six core components, with each item rated on a five-point Likert scale ranging from strongly agree to strongly disagree. Perceived risk was explored through six items that captured how personally vulnerable participants felt to developing breast cancer, touching on general susceptibility, awareness of risk factors, and the role of cultural or religious beliefs in attributing the cause of the disease. Perceived severity was covered by four items reflecting how seriously participants viewed BC, including its treatment implications, the personal toll it takes, and whether they believed divine intervention could resolve the illness. Perceived benefits were examined through three items looking at how strongly participants believed in the value of seeking early medical attention and the effectiveness of available treatments. Perceived barriers were addressed through twelve items that uncovered a broad range of obstacles to care-seeking, spanning emotional fears, logistical difficulties, financial hardships, and fatalistic outlooks. Perceived self-efficacy was assessed using three items that gauged how confident participants felt in their ability to take concrete steps toward checking and addressing their breast health. Finally, cues to action were captured through three items reflecting the social encouragement and religious motivations that prompted participants to seek medical care [8,18]. Following exploratory factor analysis, one item was found to have a poor factor loading and was subsequently removed, leaving a refined 30-item instrument.

### Instrument adaptation and validation

All instruments underwent a rigorous adaptation process to ensure cultural and linguistic appropriateness [19]. Before being used in the field, the questionnaire was thoroughly reviewed by a panel of oncologists, public health specialists, and psychologists, whose collective expertise helped confirm that the content was both relevant and valid. To further refine the instrument, pilot testing was carried out with a small group of patients who were not part of the main study sample. This step was invaluable in identifying any items that were unclear, difficult to understand, or poorly received. Based on the feedback gathered during this process, minor revisions were made to improve the overall readability and flow of the questionnaire [20]. The final instrument performed well statistically, with exploratory factor analysis successfully identifying five meaningful latent constructs and Cronbach's alpha values ranging from 0.809 to 0.871, reflecting good to excellent internal consistency and providing strong evidence of the instrument's reliability and construct validity.

### Data collection procedure

Data collection was carried out by trained research assistants who followed a carefully standardized protocol throughout the entire process. Each interview typically lasted between 25 and 35 min and was conducted in a private setting within the clinical environment, creating a comfortable and safe space where patients felt at ease and were more likely to respond openly and honestly. To maintain consistency, research assistants adhered to a structured approach for ordering questions, probing for deeper responses, and accurately recording what participants shared. Throughout the data collection period, supervisors conducted regular monitoring visits to observe interviews in progress and ensure that all research assistants were consistently following the protocol as intended.

### Data management and analysis

All data were initially recorded on structured paper forms before being entered into R software for analysis. To minimize the risk of input errors, double data entry was employed, and any discrepancies identified between the two entries were carefully resolved through open discussion between the data entry team and the research supervisor. Cases with incomplete responses were handled using listwise deletion, meaning that only fully completed questionnaires were retained for analysis, thereby preserving the integrity of the dataset.

Descriptive analyses were first performed to provide a clear overview of the demographic characteristics of the study sample, with results summarized using frequencies and percentages. Exploratory factor analysis was then conducted to uncover the underlying latent HBM constructs within the questionnaire. Items were retained in the final instrument if their factor loadings exceeded 0.50 and did not show cross-loadings above 0.32, ensuring that each item contributed meaningfully and uniquely to its respective construct. The internal consistency of each identified factor was subsequently evaluated using Cronbach's alpha [21]. To examine how specific HBM components related to patient delay, logistic regression analysis was performed. HBM component scores were divided into low and high categories based on the 75th percentile cutoff, and all statistical tests were evaluated at a significance level of p < 0.05.

### Ethical considerations

The Ethics Committee of Dr. M. Djamil Hospital in Padang, Indonesia, approved this study (No. DP.04.03/D.XVI.10.1/255/2026). All participants were properly informed about the study and gave signed consent to participate before any data were collected.

## Protocol validation

Overview of participant characteristics (Table 1).

**Table 1:** Overview of participant characteristics (n = 250).

Variable	f (%)
Age (years)	
<50	76 (30.4)
≥ 50	174 (69.6)
Breast cancer-related knowledge	
Good	35 (14.0)
Poor	215 (86.0)
Category of healthcare provider	
General medical doctor	162 (64.8)
Oncologist	88 (35.2)
Patient delay in healthcare provider consultation	
Yes	190 (76.0)
No	60 (24.0)

Table 1 shows that the majority of women enrolled in this study were aged 50 years and older (69.6%). Most participants demonstrated poor knowledge about BC (86.0%). When it came to the first healthcare provider consulted, most women turned to a general medical doctor (64.8%), while 35.2% sought care directly from an oncologist. The majority of participants (76.0%) experienced patient delay in seeking healthcare consultation.

**Fig. 1:**
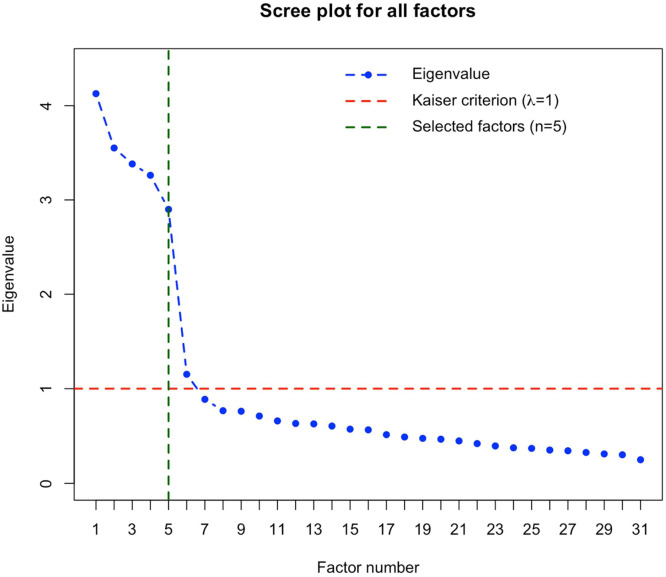
Scree plot obtained from the Health Belief Model items exploratory factor analysis.

Factor loadings on health belief model questions before item removal (Table 2 and Fig. 1).

**Table 2:** Factor loadings on health belief model questions before item removal.

Items	RC1	RC2	RC3	RC4	RC5	Decision^a^
PR1	−0.06	0.00	0.03	−0.04	0.74*	Retained
PR2	−0.03	0.00	0.00	−0.01	0.63*	Retained
PR3	−0.07	−0.09	−0.03	−0.07	0.64*	Retained
PR4	−0.07	−0.09	−0.03	0.09	0.57*	Retained
PR5	0.09	−0.01	−0.02	−0.08	0.69*	Retained
PR6	−0.01	−0.01	−0.05	0.09	0.61*	Retained
PD1	0.04	0.10	−0.10	0.69*	−0.05	Retained
PD2	0.02	−0.01	0.01	0.71*	0.05	Retained
PD3	−0.07	0.00	0.00	0.66*	0.09	Retained
PD4	0.07	−0.05	−0.09	0.68*	0.00	Retained
PBT1	0.04	0.01	0.07	0.68*	−0.04	Retained
PBT2	0.01	0.04	0.03	0.00	−0.02	Removed
PBT3	0.03	0.65*	0.03	0.00	−0.02	Retained
PB1	0.06	0.68*	−0.10	0.05	−0.09	Retained
PB2	−0.01	0.72*	−0.01	0.00	−0.01	Retained
PB3	0.00	0.67*	−0.03	0.04	−0.01	Retained
PB4	0.08	0.81*	−0.01	−0.02	−0.04	Retained
PB5	0.01	0.72*	−0.01	0.00	−0.06	Retained
PB6	0.63*	0.02	−0.07	0.08	−0.05	Retained
PB7	0.75*	0.02	−0.02	0.06	−0.07	Retained
PB8	0.78*	0.08	0.03	−0.01	0.04	Retained
PB9	0.76*	0.02	0.01	0.00	0.00	Retained
PB10	0.80*	−0.05	−0.09	0.03	−0.04	Retained
PB11	0.66*	0.08	0.09	−0.04	−0.07	Retained
PB12	0.04	−0.06	0.73*	−0.02	0.02	Retained
PS1	−0.04	0.00	0.68*	0.00	−0.05	Retained
PS2	−0.03	−0.03	0.70*	−0.07	−0.11	Retained
PS3	0.01	−0.06	0.66*	0.00	0.06	Retained
A1	−0.02	−0.04	0.70*	−0.02	−0.07	Retained
A2	0.00	0.06	0.69*	0.05	0.03	Retained
A3	−0.03	−0.02	−0.01	0.58*	−0.02	Retained

a Question items with factor loading above the threshold of 0.50 or below the threshold of −0.50 without cross-loading of >0.32 or less than −0.32 were retained; RC, rotated component; PR, perceived risk of BC; PD, perceived severity of BC; PBT, perceived benefits of BC diagnosis and treatment; PB, perceived barriers of BC treatment; PS, perceived self-efficacy; A, cues to action.

Table 2 presents the initial factor loadings of the 31 HBM items across five rotated components before any item was removed. Overall, the results painted a clear and well-defined factor structure, with most items loading strongly and cleanly onto the components they were designed to measure. Items capturing how personally at-risk participants felt of developing BC (PR1-PR6) naturally grouped together under RC5, with loadings ranging from 0.57 to 0.74. Those reflecting how seriously participants viewed the disease and how much they believed in the benefits of early diagnosis and treatment (PD1-PD4, PBT1, PBT3) fell under RC4, with loadings between 0.65 and 0.71. Items tapping into the practical obstacles that stood in the way of seeking BC treatment (PB1-PB5) clustered under RC2, with loadings ranging from 0.67 to 0.81, while those capturing financial and emotional barriers (PB6-PB11) sat under RC1, with loadings between 0.63 and 0.80. Items reflecting how confident participants felt in their ability to take action (PS1-PS3) and what prompted them to seek care (A1-A2, PB12) loaded onto RC3, with loadings ranging from 0.66 to 0.73. One item proved to be the exception, PBT2 ("The doctor can save my life"), which simply did not load meaningfully onto any single factor, achieving a maximum loading of just 0.04, far below the 0.50 retention threshold, and was consequently removed from the instrument. The remaining 30 items were taken forward into subsequent analyses, forming a refined and psychometrically robust tool for capturing HBM constructs among breast cancer patients.

Scree plot obtained from the Health Belief Model items exploratory factor analysis (Fig. 1).

Exploratory factor analysis of BC-related health belief model components (Table 3).

**Table 3:** Exploratory factor analysis of BC-related health belief model components.

Items	Factor and item statement	Item loadings	SS loading rotated	Eigenvalue unrotated	Variance explained	Cumulative variance (%)	Cronbach’s alpa (95% CI)
RC1	**Financial, emotional and access barriers**		3.261	4.099	10.9%	10.9%	0.871 (0.847–0.896)
PB6	I am unable to pay for a medical examination.	0.632					
PB7	I am afraid to find out if I have cancer.	0.749					
PB8	I'm not sure where to go to see if my breasts have changed.	0.774					
PB9	If someone had accompanied me, I would have gone for medical checkup earlier.	0.758					
PB10	A medical examination might be uncomfortable.	0.803					
PB11	I am unable to cover the cost of my breast cancer therapy.	0.656					
RC2	**Treatment beliefs and practical barriers**		3.083	3.372	10.3%	21.1%	0.846 (0.817–0.876)
PBT3	Breast cancer is a curable disease.	0.650					
PB1	I am afraid of medical checkup.	0.677					
PB2	I should not decide myself about the severity of symptoms.	0.721					
PB3	Alternative treatment will heal the symptoms.	0.673					
PB4	I have no time to go for medical checkup.	0.809					
PB5	I am unable to make travel arrangements for a medical examination.	0.716					
RC3	**Self-efficacy and social cues to action**		2.939	3.536	9.8%	30.9%	0.857 (0.830–0.885)
PB12	No matter what, if an illness is meant to happen, it will.	0.730					
PS1	I am able to attend a medical examination.	0.680					
PS2	I am willing to talk to doctor about my concerns.	0.699					
PS3	Changes in my breasts could be a serious issue, so I want to get checked out.	0.658					
A1	I was encouraged to see a doctor by my family and those around me.	0.696					
A2	Someone I know with breast cancer encouraged me to get a breast checkup.	0.689					
RC4	**Perceived severity, treatment benefits, and religious beliefs**		2.386	2.843	8.0%	47.5%	0.809 (0.772–0.847)
PD1	If breast cancer is detected early, treatment is possible.	0.696					
PD2	Breast cancer can lead to needing chemotherapy or radiotherapy treatment.	0.694					
PD3	All my life changed after I got breast cancer.	0.672					
PD4	I believe that God's will, will cause this illness to disappear.	0.664					
PBT1	Breast cancer can be treated more effectively if go to doctor early.	0.679					
A3	If I receive breast therapy, I will be adhering to a sunnah.	0.749					
RC5	**Perceived susceptibility and cultural beliefs**		2.593	2.909	8.6%	39.6%	0.811 (0.775–0.847)
PR1	I had a probability of developing breast cancer before receiving a diagnosis.	0.735					
PR2	Anyone can get breast cancer.	0.634					
PR3	I can develop breast cancer even without clear risk factors, because breast cancer can be influenced by genetic, hormonal, reproductive, lifestyle, and environmental factors.	0.646					
PR4	I was destined to have breast cancer.	0.567					
PR5	People who don't follow religion will get breast cancer	0.690					
PR6	Evil eye can be a cause of breast cancer	0.607					

Table 3 shows the results of the exploratory factor analysis of the 30 retained HBM items, which yielded five distinct factors collectively explaining 47.5% of the total variance. All five factors demonstrated good to excellent internal consistency, with Cronbach's alpha values ranging from 0.809 to 0.871.

The first factor, RC1 (Financial, emotional and access barriers), comprised six items (PB6–PB11) and accounted for the largest proportion of variance (10.9%), with an eigenvalue of 4.099 and item loadings ranging from 0.632 to 0.803. This factor captured a broad range of obstacles to seeking BC care, spanning financial constraints, emotional fears, limited knowledge of where to seek help, and the need for social accompaniment.

The second factor, RC2 (Treatment beliefs and practical barriers), consisted of six items (PBT3, PB1–PB5) and explained 10.3% of the variance, with an eigenvalue of 3.372 and item loadings ranging from 0.650 to 0.809. This factor reflected a combination of beliefs about treatment curability and practical day-to-day barriers such as time constraints, transportation difficulties, and preference for alternative treatments.

The third factor, RC3 (Self-efficacy and social cues to action), comprised six items (PB12, PS1–PS3, A1–A2) and accounted for 9.8% of the variance, with an eigenvalue of 3.536 and item loadings ranging from 0.658 to 0.730. This factor captured participants' confidence in their ability to seek care, the influence of social networks, and fatalistic beliefs about the inevitability of illness.

The fourth factor, RC4 (Perceived severity, treatment benefits, and religious beliefs), consisted of six items (PD1–PD4, PBT1, A3) and explained 8.0% of the variance, with an eigenvalue of 2.843 and item loadings ranging from 0.664 to 0.749. This factor reflected perceptions of the seriousness of BC, beliefs in the effectiveness of early treatment, and religious motivations for seeking care.

The fifth factor, RC5 (Perceived susceptibility and cultural beliefs), comprised six items (PR1–PR6) and accounted for 8.6% of the variance, with an eigenvalue of 2.909 and item loadings ranging from 0.567 to 0.735. This factor captured participants' sense of personal vulnerability to BC, alongside deeply held cultural and religious attributions of the disease, including fatalistic and supernatural explanations.

Association between components of the health belief model and patient delay among BC patients (Table 4).

**Table 4:** Association between components of the health belief model and patient delay among BC patients.

Item	No delay	Delay	Crude OR (95% CI)	Adjusted OR Model 2 (95% CI)^b^	Adjusted OR Model 3 (95% CI)^c^
Financial, emotional and access barriers					
Low	41 (29.9)	96 (70.1)	*Reference*	*Reference*	*Reference*
High	19 (16.8)	94 (83.2)	**2.13 (1.14, 3.90)**	**2.26 (1.21, 4.23)**	**2.21 (1.18, 4.16)**
Self-efficacy and social cues to action					
Low	46 (24.9)	139 (75.1)	*Reference*	*Reference*	*Reference*
High	14 (21.5)	51 (78.5)	1.21 (0.61, 2.38)	1.39 (0.69, 2.81)	1.41 (0.69, 2.85)
Treatment beliefs and practical barriers					
Low	40 (22.5)	138 (77.5)	*Reference*	*Reference*	*Reference*
High	20 (27.8)	52 (72.2)	0.75 (0.40, 1.41)	0.80 (0.42, 1.53)	0.82 (0.42, 1.58)
Perceived susceptibility and cultural beliefs					
Low	51 (28.5)	128 (71.5)	*Reference*	*Reference*	*Reference*
High	9 (12.7)	62 (87.3)	**2.75 (1.27, 5.93)**	**3.05 (1.39, 6.73)**	**3.03 (1.37, 6.68)**
Perceived severity, treatment benefits, and religious beliefs					
Low	44 (24.6)	135 (75.4)	*Reference*	*Reference*	*Reference*
High	16 (22.5)	55 (77.5)	1.12 (0.58, 2.15)	1.21 (0.62, 2.38)	1.23 (0.62, 2.42)

CI, confidence interval; OR, odds ratio.

a Items category divisions: Low = <75th percentile; High = ≥75th percentile.

b Adjusted for other health belief model components.

c Adjusted for other health belief model components, age, BC-related knowledge, category of healthcare provider.

Table 4 presents findings showing that two HBM components stood out as significantly linked to patient delay across all three models. The first was financial, emotional and access barriers, where participants who scored high on this component were found to be more than twice as likely to experience delay in seeking care compared to those who scored low, and this association held firm even after full adjustment (crude OR: 2.13, 95% CI: 1.14–3.90; adjusted OR Model 2: 2.26, 95% CI: 1.21–4.23; adjusted OR Model 3: 2.21, 95% CI: 1.18–4.16). The second, and perhaps most striking, was perceived susceptibility and cultural beliefs, which emerged as the strongest driver of patient delay among all five components. Participants with high scores on this component were approximately three times more likely to delay seeking care than those with low scores, a finding that remained robust and statistically significant even after accounting for all confounders (crude OR: 2.75, 95% CI: 1.27–5.93; adjusted OR Model 2: 3.05, 95% CI: 1.39–6.73; adjusted OR Model 3: 3.03, 95% CI: 1.37–6.68).

## Limitations

This study has several limitations that should be kept in mind when interpreting its findings. First, the cross-sectional nature of the study means that it is not possible to draw conclusions about cause and effect between health beliefs and patient delay. A longitudinal approach would have been better suited to tracking how these beliefs change over time and understanding their lasting influence on the decision to seek care. Future longitudinal studies are therefore needed to clarify the temporal relationship between HBM constructs and patient delay. Second, because the study relied entirely on self-reported data, there is an inherent risk of recall bias and social desirability bias, particularly when participants were asked to remember exactly when they first noticed their symptoms and when they eventually decided to seek help, which could affect the reliability of the patient delay estimates. Third, although the HBM questionnaire was carefully adapted and validated for the local context, it may not have been able to fully capture the rich and nuanced tapestry of cultural, spiritual, and community-level influences that shape how women in West Sumatra approach their health. Further qualitative research may help explore these deeper sociocultural meanings and strengthen future versions of the instrument. Finally, by focusing exclusively on health belief constructs, the study did not take into account other potentially important drivers of patient delay, such as barriers within the healthcare system itself, challenges related to healthcare providers, or the powerful role that family members often play in health-related decisions, all of which carry particular significance in a collectivist society like Indonesia. In addition, convenience sampling from two hospitals may restrict the transferability of the findings to wider breast cancer populations in Indonesia, especially women in rural communities or outside hospital-based care. Further testing, including confirmatory factor analysis across diverse Indonesian settings, is recommended.

## Declaration of generative AI and AI-assisted technologies in the writing process

During the development of this manuscript, Grammarly and ChatGPT were used to support improvements in clarity and readability. The authors subsequently reviewed, edited, and verified the content as appropriate and take full responsibility for the final version of the publication.

## CRediT authorship contribution statement

**Ricvan Dana Nindrea:** Conceptualization, Formal analysis, Investigation, Methodology, Software, Writing – original draft. **Armaita:** Validation, Writing – review & editing. **Ainil Mardiah:** Validation, Writing – review & editing. **Long Chiau Ming:** Validation, Writing – review & editing. **Ismeldi Syarief:** Validation, Writing – review & editing. **Muhammad Syarif Khairullah Suryadharma Putra:** Investigation. **Nadyne Falisha:** Investigation. **Regita Ayu Cahyani:** Investigation. **Tsania Faiza Mardhatillah:** Investigation. **Novia Elsaputri:** Investigation.

## Declaration of competing interest

The authors declare that they have no known competing financial interests or personal relationships that could have appeared to influence the work reported in this paper.

## Data Availability

Data will be made available on request.

## Appendix Supplementary materials

Image, application 1
