# Supplementary material for: Assessing health beliefs and patient delay among breast cancer patients in West Sumatra, Indonesia
Source: MethodsX. 2026 Jul 7;17:104035. doi: 10.1016/j.mex.2026.104035 (PMC13380172; doi:10.1016/j.mex.2026.104035)
Supplement: Supplementary file 1 [file mmc1.docx]

**QUESTIONNAIRE**

**ASSESSING HEALTH BELIEFS AND PATIENT DELAY AMONG BREAST CANCER PATIENTS IN WEST SUMATRA, INDONESIA**

Date: dd/mm/yyyy

**Section A. Participant characteristics**

| **No** | **Questions** | **Answer (Please tick)** |
| --- | --- | --- |
| A1 | Unique ID | [ ] [ ] [ ] [ ] |
| A2 | Age | ______years |
| A3 | Maternal education level | [ ] 1. No formal schooling  [ ] 2. Primary education  [ ] 3. Junior high school  [ ] 4. Higher education (college/university) |
| A4 | Who was the first healthcare provider you consulted when you first noticed changes in your breast? | [ ] 1. General medical doctor  [ ] 2. Oncologist |

**Section B. Patient delay in healthcare provider consultation**

Please answer the following questions as accurately as possible.

| **No** | **Questions** | **Answer** |
| --- | --- | --- |
| B1 | When did you first notice any changes or symptoms in your breast? | Month: ____ Year: ____ |
| B2 | When did you first feel that these changes needed medical attention? | Month: ____ Year: ____ |
| B3 | When did you first consult a healthcare provider about these changes? | Month: ____ Year: ____ |
| B4 | How long did it take from when you first noticed the changes to when you first sought medical attention? | ____ days / ____ weeks / ____ months |

**Section C. Health beliefs**

The Health Beliefs section consists of a 31-item questionnaire. Participants are asked to respond to each question based on their personal beliefs and perceptions.

| **No** | **Questions** | **Answer (Please tick)** |
| --- | --- | --- |
| **PR** | **Perceived risk of breast cancer** |  |
| PR1 | Before getting diagnosed, I had a chance to get breast cancer | [ ] 1. Strongly disagree  [ ] 2. Disagree  [ ] 3. Not sure  [ ] 4. Agree  [ ] 5. Strongly agree |
| PR2 | Anyone can get breast cancer. | [ ] 1. Strongly disagree  [ ] 2. Disagree  [ ] 3. Not sure  [ ] 4. Agree  [ ] 5. Strongly agree |
| PR3 | I can develop breast cancer even without clear risk factors, because breast cancer can be influenced by genetic, hormonal, reproductive, lifestyle, and environmental factors. | [ ] 1. Strongly disagree  [ ] 2. Disagree  [ ] 3. Not sure  [ ] 4. Agree  [ ] 5. Strongly agree |
| PR4 | I got breast cancer because it is my destiny | [ ] 1. Strongly disagree  [ ] 2. Disagree  [ ] 3. Not sure  [ ] 4. Agree  [ ] 5. Strongly agree |
| PR5 | People who don't follow religion will get breast cancer | [ ] 1. Strongly disagree  [ ] 2. Disagree  [ ] 3. Not sure  [ ] 4. Agree  [ ] 5. Strongly agree |
| PR6 | Evil eye can be a cause of breast cancer | [ ] 1. Strongly disagree  [ ] 2. Disagree  [ ] 3. Not sure  [ ] 4. Agree  [ ] 5. Strongly agree |
| PD | **Perceived severity of breast cancer** |  |
| PD1 | Breast cancer can be treated if the disease is in early stage | [ ] 1. Strongly disagree  [ ] 2. Disagree  [ ] 3. Not sure  [ ] 4. Agree  [ ] 5. Strongly agree |
| PD2 | Breast cancer can lead to needing chemotherapy or radiotherapy treatment. | [ ] 1. Strongly disagree  [ ] 2. Disagree  [ ] 3. Not sure  [ ] 4. Agree  [ ] 5. Strongly agree |
| PD3 | All my life changed after I got breast cancer. | [ ] 1. Strongly disagree  [ ] 2. Disagree  [ ] 3. Not sure  [ ] 4. Agree  [ ] 5. Strongly agree |
| PD4 | I think this disease will go away by the will of God. | [ ] 1. Strongly disagree  [ ] 2. Disagree  [ ] 3. Not sure  [ ] 4. Agree  [ ] 5. Strongly agree |
| **PBT** | **Perceived benefits of breast cancer diagnosis and treatment** |  |
| PBT1 | Breast cancer can be treated more effectively if go to doctor early. | [ ] 1. Strongly disagree  [ ] 2. Disagree  [ ] 3. Not sure  [ ] 4. Agree  [ ] 5. Strongly agree |
| PBT2 | The doctor can save my life. | [ ] 1. Strongly disagree  [ ] 2. Disagree  [ ] 3. Not sure  [ ] 4. Agree  [ ] 5. Strongly agree |
| PBT3 | Breast cancer is a curable disease. | [ ] 1. Strongly disagree  [ ] 2. Disagree  [ ] 3. Not sure  [ ] 4. Agree  [ ] 5. Strongly agree |
| **PB** | **Perceived barriers of breast cancer treatment** |  |
| PB1 | I am afraid of medical checkup. | [ ] 1. Strongly disagree  [ ] 2. Disagree  [ ] 3. Not sure  [ ] 4. Agree  [ ] 5. Strongly agree |
| PB2 | I should not decide myself about the severity of symptoms. | [ ] 1. Strongly disagree  [ ] 2. Disagree  [ ] 3. Not sure  [ ] 4. Agree  [ ] 5. Strongly agree |
| PB3 | Alternative treatment will heal the symptoms. | [ ] 1. Strongly disagree  [ ] 2. Disagree  [ ] 3. Not sure  [ ] 4. Agree  [ ] 5. Strongly agree |
| PB4 | I have no time to go for medical checkup. | [ ] 1. Strongly disagree  [ ] 2. Disagree  [ ] 3. Not sure  [ ] 4. Agree  [ ] 5. Strongly agree |
| PB5 | I cannot arrange transportation to get a medical checkup. | [ ] 1. Strongly disagree  [ ] 2. Disagree  [ ] 3. Not sure  [ ] 4. Agree  [ ] 5. Strongly agree |
| PB6 | I cannot afford to pay for medical checkup. | [ ] 1. Strongly disagree  [ ] 2. Disagree  [ ] 3. Not sure  [ ] 4. Agree  [ ] 5. Strongly agree |
| PB7 | I am afraid to find out if I have cancer. | [ ] 1. Strongly disagree  [ ] 2. Disagree  [ ] 3. Not sure  [ ] 4. Agree  [ ] 5. Strongly agree |
| PB8 | I do not know where to go if I want to check for changes in my breast. | [ ] 1. Strongly disagree  [ ] 2. Disagree  [ ] 3. Not sure  [ ] 4. Agree  [ ] 5. Strongly agree |
| PB9 | If someone had accompanied me, I would have gone for medical checkup earlier. | [ ] 1. Strongly disagree  [ ] 2. Disagree  [ ] 3. Not sure  [ ] 4. Agree  [ ] 5. Strongly agree |
| PB10 | Medical checkup can be painful. | [ ] 1. Strongly disagree  [ ] 2. Disagree  [ ] 3. Not sure  [ ] 4. Agree  [ ] 5. Strongly agree |
| PB11 | I cannot afford to pay for the treatment of breast cancer. | [ ] 1. Strongly disagree  [ ] 2. Disagree  [ ] 3. Not sure  [ ] 4. Agree  [ ] 5. Strongly agree |
| PB12 | If a disease is destined to happen, it will occur, no matter what. | [ ] 1. Strongly disagree  [ ] 2. Disagree  [ ] 3. Not sure  [ ] 4. Agree  [ ] 5. Strongly agree |
| **PS** | **Perceived self-efficacy** |  |
| PS1 | I can manage to go for a medical checkup. | [ ] 1. Strongly disagree  [ ] 2. Disagree  [ ] 3. Not sure  [ ] 4. Agree  [ ] 5. Strongly agree |
| PS2 | I am willing to talk to doctor about my concerns. | [ ] 1. Strongly disagree  [ ] 2. Disagree  [ ] 3. Not sure  [ ] 4. Agree  [ ] 5. Strongly agree |
| PS3 | I want to have a breast checkup because changes in my breast may be something serious. | [ ] 1. Strongly disagree  [ ] 2. Disagree  [ ] 3. Not sure  [ ] 4. Agree  [ ] 5. Strongly agree |
| **A** | **Cues to action** |  |
| A1 | My family or people around me encouraged me to go to doctor | [ ] 1. Strongly disagree  [ ] 2. Disagree  [ ] 3. Not sure  [ ] 4. Agree  [ ] 5. Strongly agree |
| A2 | Someone I know with breast cancer encouraged me to get a breast checkup. | [ ] 1. Strongly disagree  [ ] 2. Disagree  [ ] 3. Not sure  [ ] 4. Agree  [ ] 5. Strongly agree |
| A3 | I will be following a sunnah if I go for breast treatment. | [ ] 1. Strongly disagree  [ ] 2. Disagree  [ ] 3. Not sure  [ ] 4. Agree  [ ] 5. Strongly agree |
